# Evaluation of genetic relationship among varieties of *Capsicum annuum* L. and *Capsicum frutescens* L. in West Africa using ISSR markers

**DOI:** 10.1016/j.heliyon.2019.e01700

**Published:** 2019-05-14

**Authors:** Tomi Lois Olatunji, Anthony Jide Afolayan

**Affiliations:** Medicinal Plants and Economic Development (MPED) Research Centre, Department of Botany, University of Fort Hare, Alice, 5700, South Africa

**Keywords:** Genetics, Molecular biology, Plant biology

## Abstract

The taxonomic identity of two closely related *Capsicum* species; *Capsicum annuum* and *Capsicum frutescens* in West Africa has not been clarified because they have overlapping morphological traits. Effective control and management measures as well as improvement of crop plants in any breeding programme can only be implemented when plant species are correctly identified. The genetic relationships of the varieties of these *Capsicum* species were assessed using 10 ISSR primers for the first time. The varieties and species used include *C. annuum* var. *abbreviatum*; *C. annuum* var. *acuminatum*; *C. annuum* var. *grossum* and *C. frutescens* var. *baccatum.* PCR amplification of the isolated DNA from the four varieties of *Capsicum* revealed a total of 75 loci out of which 14 were found to be polymorphic. Average polymorphism information content (PIC) and heterozygosity (He) of the 10 ISSR markers were estimated as 0.67 and 0.78 respectively. The relatedness among the varieties assessed by Unweighted Pair Group Method with Arithmetic Mean (UPMGA) cluster analysis did not separate *C. frutescens* var. *baccatum* from the three cultivated varieties of *C. annuum*. The result from the principal component analysis (PCA) further supports the genetic relatedness and groupings obtained from the cluster analysis. Overall, the study indicated that ISSR markers were effective in assessing the genetic relatedness and revealed genetic homogeneity of the four varieties. Our results, therefore, support the inclusion of *C. frutescens* var. *baccatum* as a variety of *C. annuum* species.

## 1 Introduction

The genus *Capsicum*, commonly known as chili or pepper belongs to the family *Solanaceae* (Bosland and Votava, 2000). They are important vegetable and spice that are cultivated in the tropical and subtropical regions of the world. *Capsicum* species are immensely valued not only because of their economic importance but also for their rich nutritional value. Besides the nutritional benefits of pepper and their use as food additives, the hot *Capsicum* species (due to their capsaicin content) have a significant role in pharmacy and are currently used for different therapeutic purposes (Xiao-min et al., 2016).

Approximately, the genus *Capsicum* consists of 35 species out of which five are widely domesticated. These are *C. annuum* L*., C. chinenses* Jacqs.*, C. frutescens* L*., C. pubescens* R*.* and *C. baccatum* L (Garcia et al., 2016). *Capsicum* spp. are diploids, mostly having 24 chromosomes (*n* = *x* = 12), and numerous wild species consisting of 26 chromosomes (*n* = *x* = 13). The domesticated species belong to the first group (Tong and Bosland, 2003).

In West Africa, the genus is represented by two cultivated species; *C*. *annuum* and *C*. *frutescens* with four main varieties. However, the taxonomic identity of these species has not been clarified because they have overlapping morphological traits. A persistent question in their taxonomy is whether these cultivated species are two different species or botanical varieties of the same species. There are varieties that possess one or more diagnostic morphological characters from one species and the rest of the characters from the other species thus, creating difficulties in species assignment. A number of adverse reactions have been reported globally because of erroneous identification and classification of plants with medicinal importance (Chen et al., 2014).

Over the years, identification and classification of the cultivated *Capsicum* species are based mainly on morphological, chemical and anatomical descriptors (Ince e*t al*., 2010). However, these methods have their limitations especially the impact of environment on phenotype, making classification phenetic rather than phylogenetic. Also, evaluation of plant materials on the field for classification is time-consuming and laborious, especially when evaluating a large number of accessions.

Considering the limitations of morphological characterization, molecular markers have been recognized as valuable tools that allow characterization of genotypes and precise measurement of the extent of genetic relatedness and dissimilarity in different plant species (Karaca and Onus, 2010; Subramanyam et al., 2012; Chen et al., 2014; Prasad, 2014). A number of DNA based molecular markers have been developed for the determination of phylogenetic relatedness within and among plant species. Inter Simple Sequence Repeat (ISSR) is one of the most widely used DNA-based markers that has been effectively used in elucidating the genetic variation and relatedness within and among several plant species (Jia et al., 2011; Thul et al., 2012; Animasaun et al., 2015; Sunar et al., 2016; Igwe et al., 2017). They are reproducible, highly polymorphic, independent of environmental influence, cost-effective and doesn't require prior sequence knowledge (Jia et al., 2011; Thul et al., 2012; Animasaun et al., 2015; Sunar et al., 2016; Igwe et al., 2017). A correct botanical classification and identification is a basic step in any improvement programme. This allows effective selection of parental genotypes in plant breeding programmes that are developed for various nutritional and pharmacological purposes (Chen et al., 2014; Animasaun et al., 2015).

There is dearth of information on molecular characterization of the cultivated *Capsicum* species in West Africa. The present investigation, therefore, evaluated the genetic relationship within and among the cultivated varieties of *Capsicum* species using ISSR markers to obtain a better knowledge of their species relationship.

## 2 Materials and method

The study was carried out at the molecular biology laboratory, Medicinal Plants and Economic Development (MPED) Research Unit, University of Fort Hare, Alice, South Africa.

### 2.1 Plant material

**Fig. 1:**
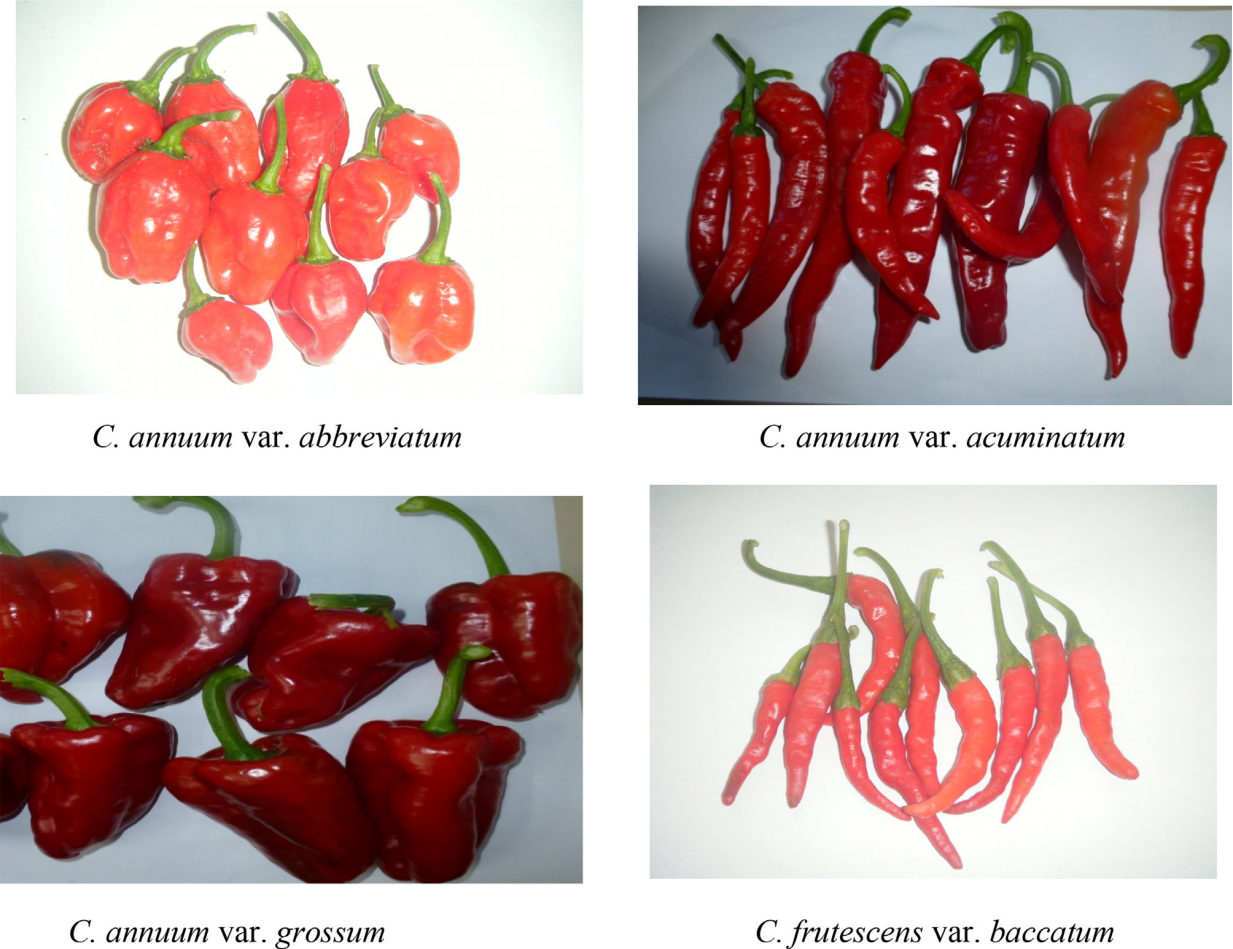
Images showing the typical shapes and sizes of the four varieties of the cultivated *Capsicum* species in West Africa assessed for the phylogenetic relationship within and among them.

Seeds of the four varieties of the cultivated *Capsicum* species (*C*. *annuum* var. *abbreviatum*, *C. annuum* var. *acuminatum*, *C. annuum* var. *grossum* and *C. frutescens* var. *baccatum*) were removed from mature fruits (Fig. 1) and grown in labeled pots in the green house of the University of Fort Hare.

### 2.2 DNA isolation and quantification

Two grams of young and fresh leaves were harvested from each plant and crushed in liquid nitrogen using pre-chilled mortar and pestle and the powder was transferred into labeled test tubes. Genomic DNA was isolated using a plant quick-DNA^TM^ miniprep kit, (Zymo Research), following the manufacturer's instructions. Quantification of DNA was done using a Nanodrop 2000 (Thermo Scientific, USA) and the purity was measured at an absorbance ratio of 260/280nm.

### 2.3 ISSR-PCR amplification

A total of 10 ISSR primers synthesized by Inqaba Biotechnical Industries (Pty) Ltd, South Africa were used (Table 1). PCR amplification was carried out in a 25 μl reaction volume containing 12.5 μl master-mix, 1 μl primers, 2.5 μl genomic DNA template and 9 μl nuclease free water. Amplification was performed in the thermocycler (Bio-Rad Mycycler USA) using the following cycling conditions: initial denaturation at 94 °C for 5 min, followed by 38 cycles of denaturation at 94 °C for 1 min, annealing at (53–64 °C) for 1 minute, extension at 72 °C for 1 min and final extension at 72 °C for 10 min.

**Table 1:** Specification of the ISSR oligonucleotides used for assessing genetic relatedness in the *Capsicum* species.

Name	Short sequence	Extended sequence (5′-3′)	Bases
Primer 1	(CT)_8_ GC	CTCTCTCTCTCTCTCTGC	18
Primer 2	(CT_8)_ G	CTCTCTCTCTCTCTCTG	17
Primer 3	CT (CCT)_5_ C	CTCCTCCTCCTCCTCCTC	18
Primer 4	(AC)_8_ T	ACACACACACACACACT	17
Primer 5	(GA)_7_ GC	GAGAGAGAGAGAGAGC	16
Primer 6	(GA)_12_	GAGAGAGAGAGAGAGAGAGAGAGA	24
Primer 7	(TC)_7_CC	TCTCTCTCTCTCTCCC	16
Primer 8	(GA)_8_T	GAGAGAGAGAGAGAGAT	17
Primer 9	(AG)_10_T	AGAGAGAGAGAGAGAGAGAGT	21
Primer10	(AG)_8_C	AGAGAGAGAGAGAGAGC	17

### 2.4 Separation and visualization of amplified products

Amplified products were separated on 1.5% w/v agarose gel stained with ethidium bromide, in 0.5X TBE buffer. 2.5 μl of each amplified sample was loaded into each well of the electrophoretic tank (BioRad, USA) and left to run for 45 minutes at 100V. Gels were photographed under UV transilluminator. Sizes of the amplified products were determined using a 1 kb molecular weight marker (O’ gene ruler, Thermo scientific, USA) as standard.

### 2.5 ISSR data analysis

The experiment was repeated twice using each primer to check for reproducibility of DNA bands. Clear bands in the size range of 250bp to 2.55kb amplified by ISSR markers were scored as (1) presence and (0) absence for each variety and were analyzed. The efficacy of each of the primer was determined using the polymorphic information content (PIC) and He. PIC values were computed based on the formula PIC = 1- ∑Pi^2^ where Pi is the frequency of the ith allele at a given locus (Anderson et al., 1993). A dendrogram showing the genetic relationship among the varieties was constructed based on the scored data, using the unweighted pair group method of arithmetic average (UPGMA). The analysis was performed using the Numerical Taxonomy and Multivariate Analysis System (NTSYSpc) V.2. software.

## 3 Results

Genomic DNA (gDNA) concentrations isolated from the four varieties of *Capsicum* species are presented in Table 2 and ranged from 114.7 ng/μl in *C. frutescens* var. *baccatum* to 216.7 ng/μl in *C. annuum* var. *abbreviatum* indicating the presence of pure DNA.

**Table 2:** Concentration and purity of gDNA isolated from the four varieties of the cultivated *Capsicum* species.

S/N	Varieties	Local names	gDNA ng/μl	^ƚ^ OD 260/280 nm
1	*C. annuum* var. *abbreviatum*	Rodo	107.5	1.83
2	*C. annuum* var. *acuminatum*	Sombo	196.9	1.85
3	*C. annuum* var. *grossum*	Tatase	216.7	1.72
4	*C. frutescens* var. *baccatum*	Wewe	114.7	1.90

### 3.1 ISSR analysis

**Fig. 2:**
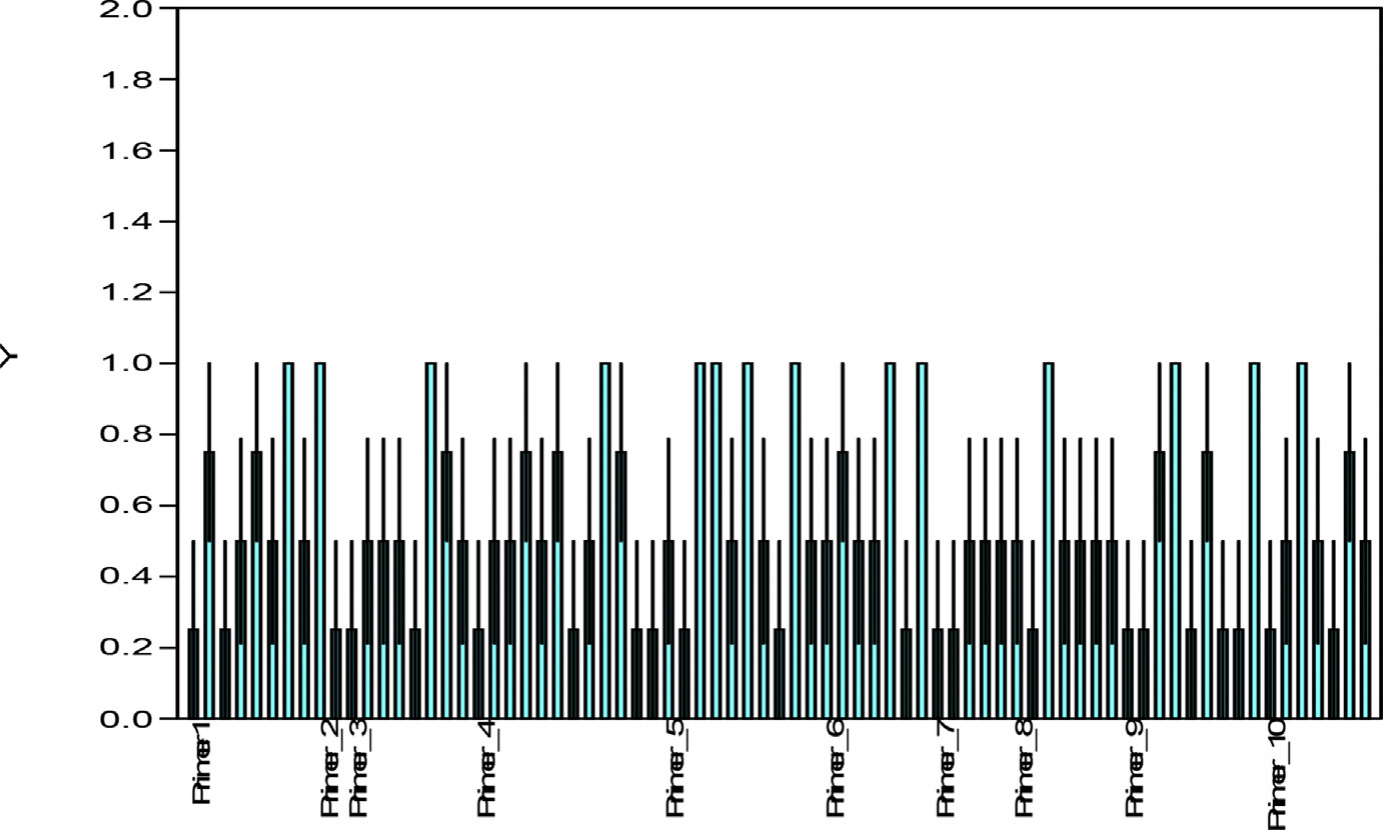
Allelic frequency amplification of the 10 ISSR primers on the four varieties of *Capsicum* species. Y-axis shows the allelic frequency while the X-axis shows the ISSR primers should for the phylogenetic relationship study.

**Fig. 3:**
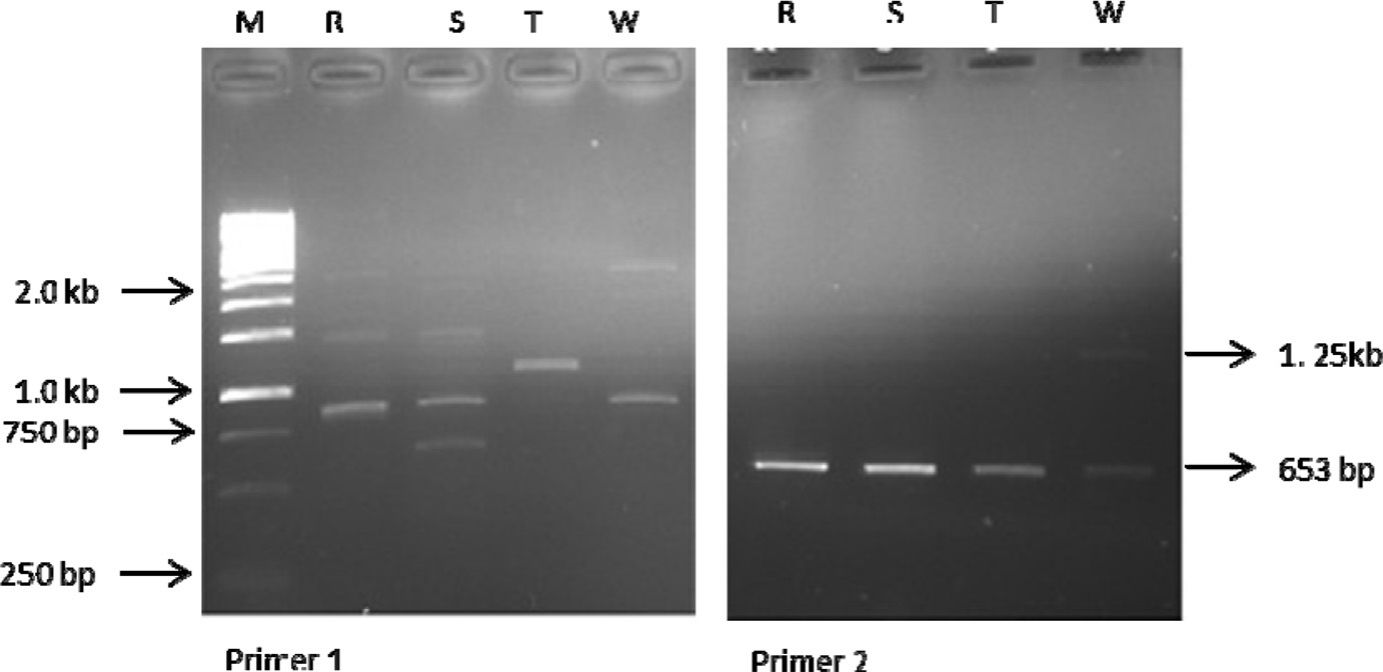
Amplification profiles of the four *Capsicum* varieties using ISSR 1&2 primers. M-molecular weight marker, R- *C. annuum* var. *abbreviatum*, S- *C. annuum* var. *acuminatum*, T- *C. annuum* var. *grossum*, W- *C. frutescens* var. *baccatum*.

**Fig. 4:**
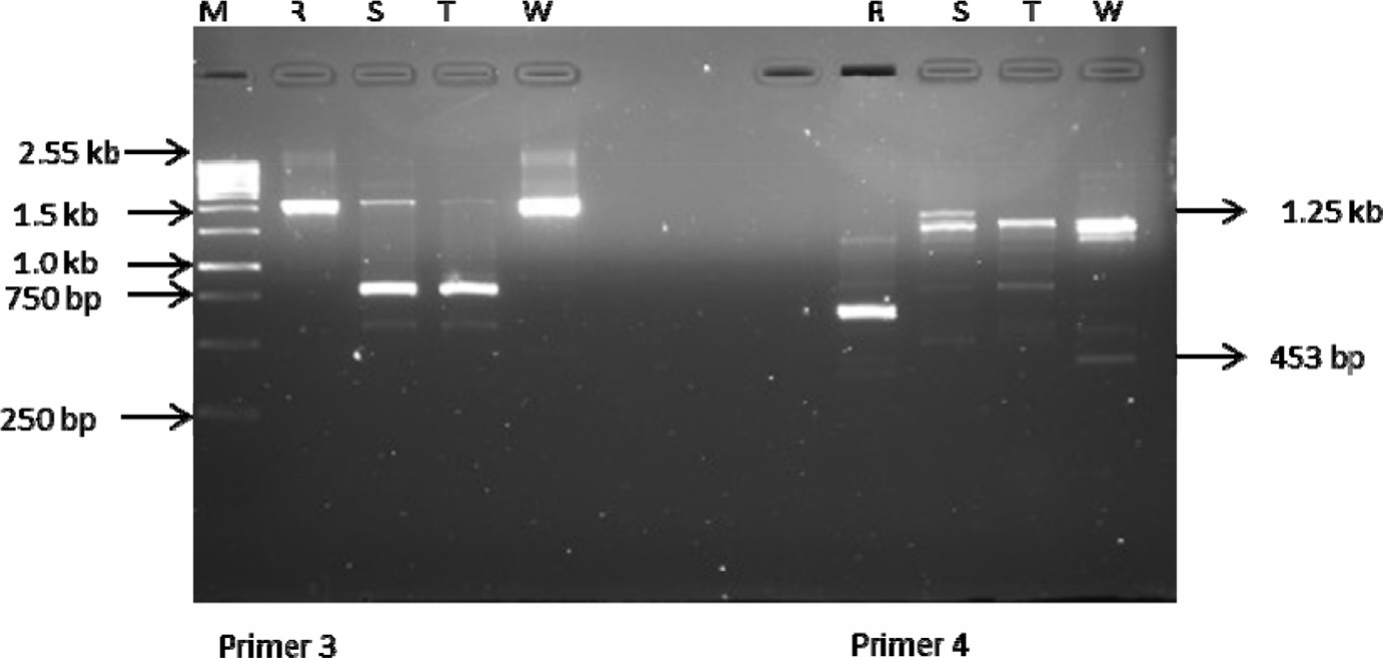
Amplification profiles of the four *Capsicum* varieties using ISSR 3 & 4 primers. M-molecular weight marker, R- *C. annuum* var. *abbreviatum*, S-*C. annuum* var. *acuminatum*, T- *C. annuum* var. *grossum*, W- *C. frutescens* var. *baccatum*.

**Fig. 5:**
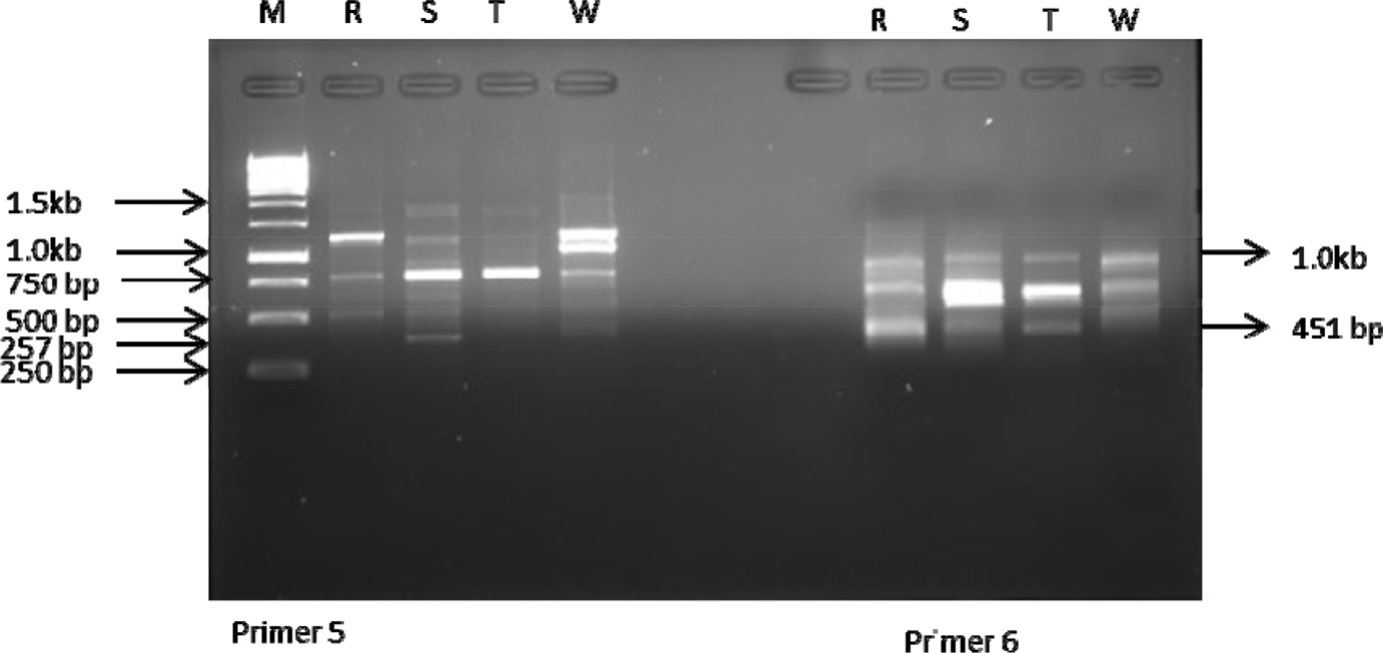
Amplification profiles of the four *Capsicum* varieties using ISSR 5 & 6 primers. M-molecular weight marker, R- *C. annuum* var. *abbreviatum*, S-*C. annuum* var. *acuminatum*, T- *C. annuum* var. *grossum*, W- *C. frutescens* var. *baccatum*.

**Fig. 6:**
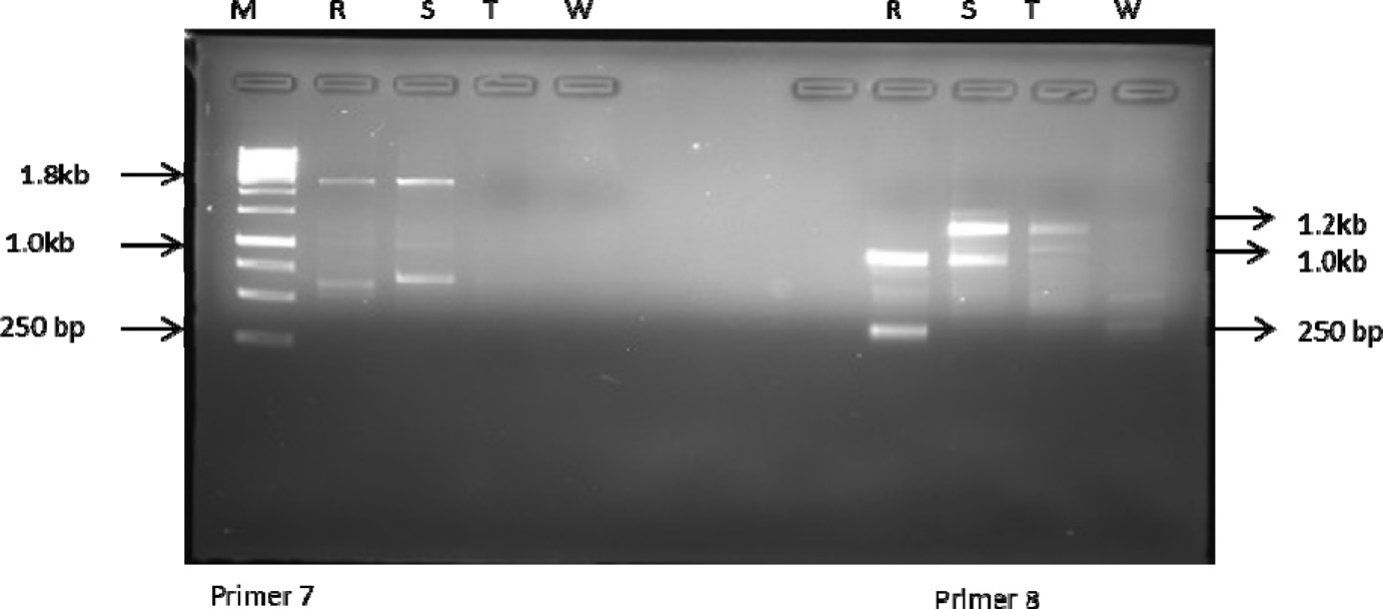
Amplification profiles of the four *Capsicum* varieties using ISSR 7 & 8 primers. M-molecular weight marker, R- *C. annuum* var. *abbreviatum*, S-*C. annuum* var. *acuminatum*, T- *C. annuum* var. *grossum*, W- *C. frutescens* var. *baccatum*.

**Fig. 7:**
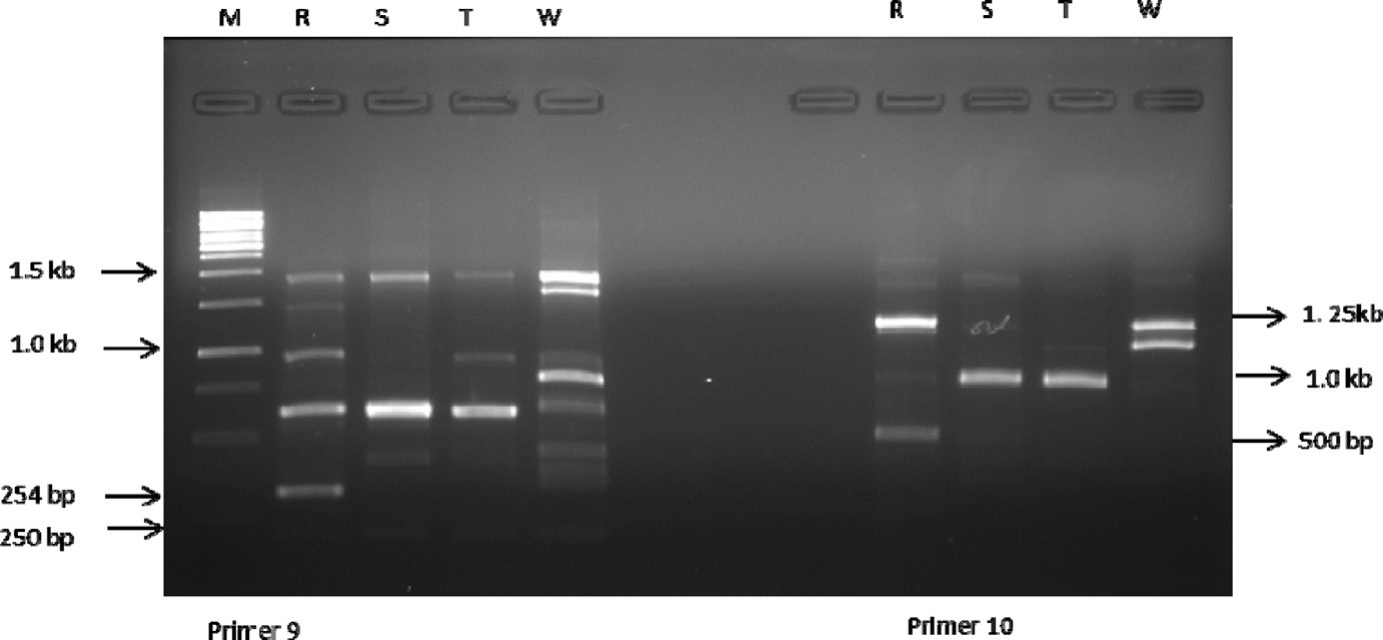
Amplification profiles of the four *Capsicum* varieties using ISSR 9 & 10 primers. M-molecular weight marker, R- *C. annuum* var. *abbreviatum*, S-*C. annuum* var. *acuminatum*, T- *C. annuum* var. *grossum*, W- *C. frutescens* var. *baccatum*.

ISSR analysis based on the 10 primers used produced a total of 75 bands out of which 14 were polymorphic. The total number of amplicons varied from 2 in primer 2 to 12 in primer 4 with an average number of 7.5 loci per primer. All primers amplified 5 and above scorable bands apart from primer 2 (Figs. 3, 4, 5, 6, and 7; Table 3). Percentage polymorphism ranged between 0 in primer 7–50 % in primer 2. The average number of polymorphic band and percentage polymorphism were 1.4 and 18.67 respectively. The allelic frequency amplification of the 10 ISSR primers used is shown in Fig. 2. Primers 4 and 5 have the highest allelic frequency of 25 and 26 respectively (Table 3). The efficacy of the markers used quantified by PIC varied from 0.27 in primer 2 to 0.87 in primer 5. Similarly, heterozygosity ranged from 0.32 in primer 2 to 0.88 in primer 5 (Table 3).

**Table 3:** Total number of amplified fragments and number of polymorphic bands generated by 10 ISSR primers in the four varieties of *Capsicum* species.

S/N	Primers (5′-3′)	Tm (°C)	TNA	TNB	NM	NP	% P	PIC	He
1	(CT)_8_GC	58	18	8	7	1	12.5	0.83	0.85
2	(CT)_8_GG	56	4	2	1	1	50	0.27	0.32
3	CT (CCT)_5_C	63	17	8	7	1	12.5	0.83	0.85
4	(AC)_8_T	53	25	12	11	1	8.33	0.69	0.75
5	(GA)_7_GC	56	26	10	6	4	40	0.87	0.88
6	(GA)_12_	64	18	7	5	2	28.57	0.81	0.83
7	(TC)_7_CC	56	7	5	5	0	0	0.74	0.77
8	(GA)_8_T	56	15	7	6	1	14.29	0.82	0.84
9	(AG)_10_T	58	19	9	7	2	22.22	0.83	0.85
10	(AG)_8_C	56	14	7	6	1	14.29	0.8	0.83
Total			164	75	61	14			
Average			16.4	7.5	6.1	1.4	18.67	0.67	0.78

Tm (°C)- Annealing temperature; TNA-Total number of Alleles; TNB- Total number of bands; NM-Number of monomorphic band; NP-Number of polymorphic band; %P- percentage polymorphism; PIC-Polymorphic information content; He- Heterozygosity.

### 3.2 Cluster analysis

**Fig. 8:**
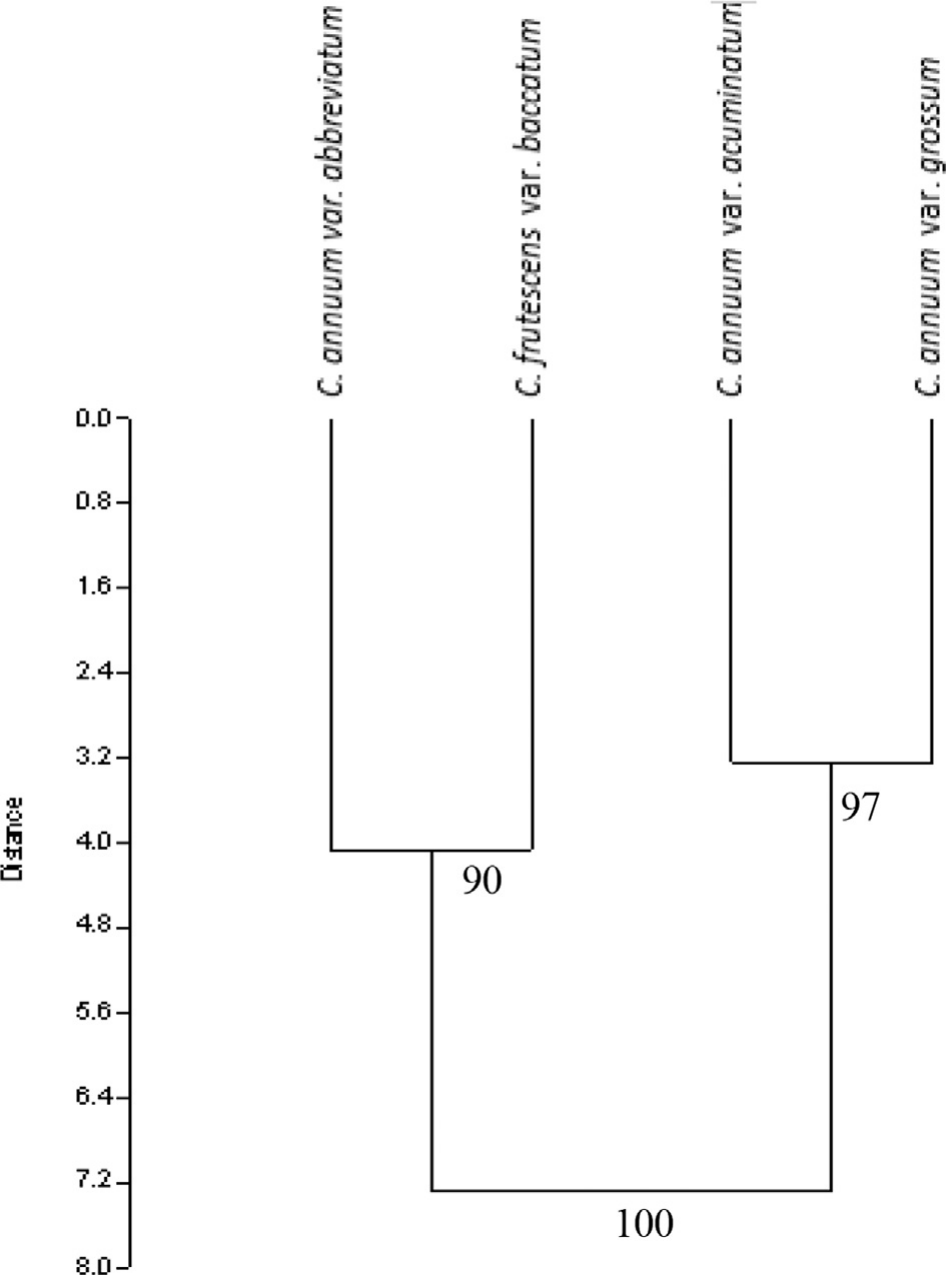
ISSR-based dendrogram of the genetic similarities among the four varieties of *Capsicum* species obtained by UPMGA.

**Fig. 9:**
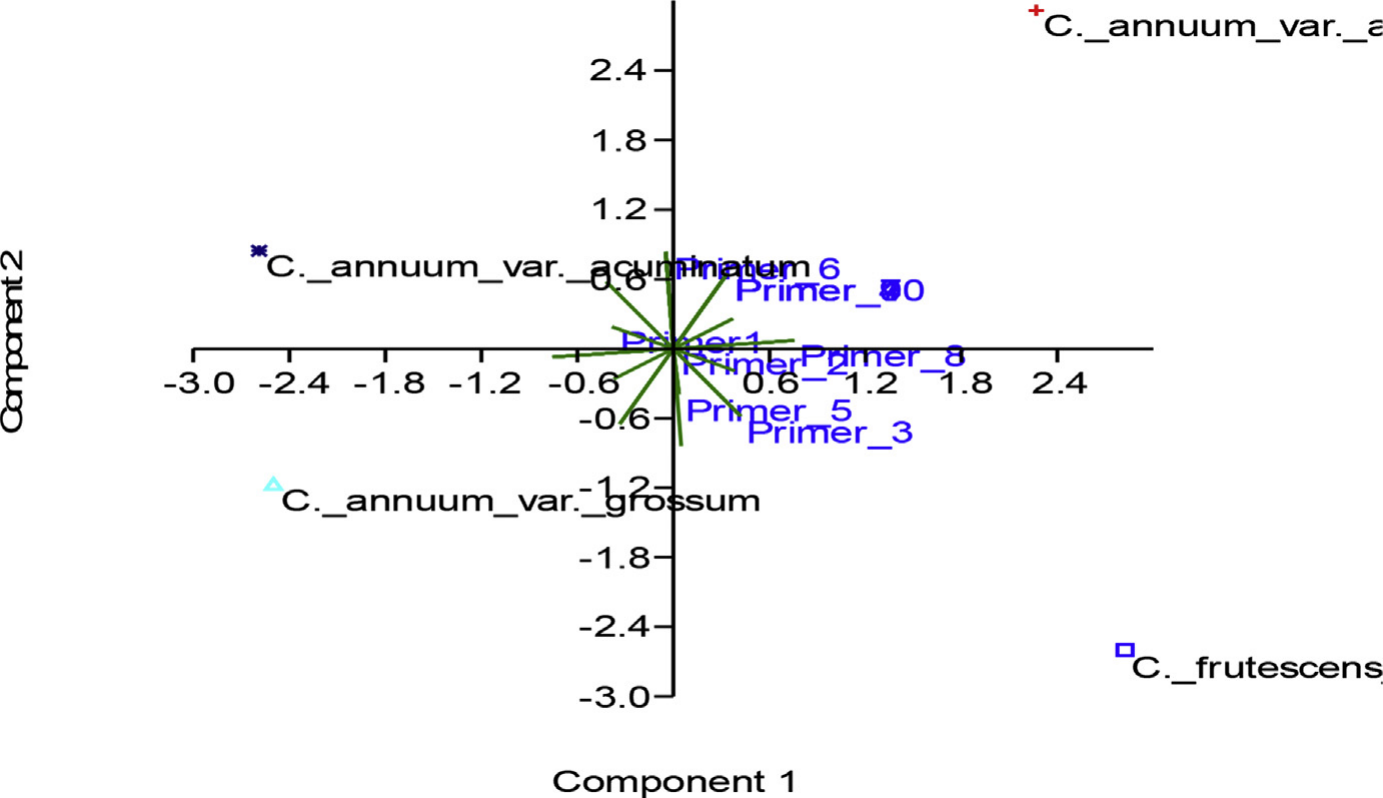
Bi-plot analysis by PCA of the four varieties of *Capsicum* species using the 10 ISSR primers.

The dendrogram constructed by UPMGA grouped the 4 varieties into 2 major clusters. Cluster 1 consists of *C*. *annuum* var. *abbreviatum* and *C. frutescens* var. *baccatum* at 90% similarity while cluster 2 consists of *C. annuum* var. *grossum* and *C. annuum* var. *acuminatum* at 97% similarity. The two clusters were connected at a similarity of 100% indicating a single lineage of the four varieties (Fig. 8). The results obtained from the principal component analysis (PCA) corroborated with that of the cluster analysis (Fig. 9) which revealed a close relationship between *C*. *annuum* var. *abbreviatum* and *C. frutescens* var. *baccatum* and also a close relationship between *C. annuum* var. *grossum* between *C. annuum* var. *acuminatum.* It also showed that seven out of the 10 primers used contributed significantly and accounted for 81.9% of microsatellite variations observed among the *Capsicum* varieties. The first component in the PCA with the largest eigenvalue (8.68488) accounted for 49.16% of the total variation, while the second component accounted for 33% of the variation with an eigenvalue of (5.78468).

## 4 Discussion

Molecular markers give accurate genetic information over biochemical, cytological and morphological markers and they help to better understand the genetic relationships between and among plant species (Ibarra-Torres et al., 2014; Patel et al., 2015). Assessment of genetic relatedness and variation is critical in effective management and improvement of crop plants (Igwe et al., 2017). In this study, the genetic relationships of the varieties of the cultivated *Capsicum* species in West Africa not previously fully investigated were evaluated using ISSR markers.

DNA amplification by PCR is dependent on the quality of genomic DNA extracted alongside several other factors. The quality of DNA is normally measured by the optical density value at an absorbance of 260/280 nm and values in the range of 1.8 and 2.0 indicate the presence of pure DNA. Lower and higher values than these indicate the presence of protein and RNA contamination respectively (Animasaun et al., 2015). In this study, the optical density values of the gDNA obtained in the four varieties used (1.72–1.90) indicated the presence of pure DNA and were effectively used for amplification.

There are several reports on the efficacy of PCR-based techniques including ISSR among other markers in evaluating the relationship or variability between different varieties of *Capsicum* (Ibarra-Torres et al., 2014). Generally, all the ISSR markers used in the study produced clear and reproducible amplification profiles. The efficacy of the markers further quantified by PIC and He values showed that the ISSR primers were effective in assessing the genetic relatedness in the varieties of the cultivated *Capsicum* species. The PIC and He values were greater than 0.2 in all the markers. This is an indication of their effectiveness (Mandal et al., 2013). Our results with respect to the efficacy of the primers based on the PIC values are consistent with the report of Rana et al. (2014) with an average PIC value of 0.60 in *C. annuum* germplasm. However, higher mean value of PIC (0.77) was reported by Ibarra-Torres et al. (2014) in their study of inter- and intraspecific differentiation of *C. annuum* and *C. pubescens* using ISSR markers.

The degree of polymorphism is an indication of the extent of genetic variation in plant species (Pfeiffer et al., 2011). The assessment of polymorphism for the ISSR primers across the four varieties revealed low genetic variation. The average percentage polymorphism was 18.67 %. This low polymorphism suggests low genetic diversity of the varieties and genetic homogeneity among the varieties. Also, the loci amplified by the primers may be adaptive genes which have become fixed in the species over evolutionary time. This result corroborates the findings of Olatunji and Morakinyo (2015) where they reported low genetic diversity among the varieties of *C. annuum* and *C. frutescens* based on their protein profiling using SDS-PAGE.

In addition to establishing the effectiveness of the ISSR markers used for profiling the DNA, the differences and similarity in the band scores revealed the genetic relatedness among the varieties of the cultivated *Capsicum* species based on UPMGA analysis. The clustering of genotypes, in this case varieties, into groups in the UPMGA -based dendrogram was based on genetic similarities and genotypes that clustered into similar groups are closely related to each other (Maity et al., 2009; Bibi et al., 2013; Tyagi et al., 2014; Dikshita and Sivarajb, 2015). The dendrogram revealed that the varieties were broadly grouped into two major clusters. *C. frutescens* var. *baccatum* did not stand out as a different species on the dendrogram but rather clustered with *C. annuum* var. *abbreviatum* at 90% similarity. Also, *C. annuum* var. *acuminatum* and *C. annuum* var. *grossum* were grouped together on the second cluster. The PCA also corroborated the result of the dendrogram; therefore relatedness among the varieties seems to be meaningful. This grouping further strengthens the proposition that *C. frutescens* and *C. annuum* maybe be varieties of a single species. Furthermore, the genetic similarity between *C. frutescens* var. *baccatum* and *C. annuum* var. *abbreviatum* was greater than the genetic similarity between the other two varieties of *C. annuum*.

Overall, the result from this study appears to harmonize with the morphological characterization (Olatunji and Afolayan, 2018), hybridization studies (Olatunji and Morakinyo, 2016), SDS-PAGE profiling (Olatunji and Morakinyo, 2015) phytochemical and antioxidant contents profiling (Olatunji and Afolayan, 2019), nutritional analysis as well as micromorphological assessment that were all used as an additional tool in understanding the genetic relatedness among the varieties of cultivated *Capsicum* species in West Africa. Whether the species concept used is phenetic (morphology, anatomy and phytochemistry) or phylogenetic, they all support the proposition that the genotypes studied are varieties of a single species- *C. annuum*.

## 5 Conclusion

The ISSR markers proved to be effective in understanding the genetic relatedness among the varieties of the cultivated *Capsicum* species investigated. The phylogenetic analysis derived from the UPMGA strengthens the proposition that the four varieties of *C. annuum* and *C. frutescens* are varieties of one species. The study, therefore, supports the inclusion of *Capsicum frutescens* var. *baccatum* as a variety of *Capsicum annuum* in West Africa. This classification however, is not applicable to the global *Capsicum* species available but the varieties studied in West Africa.

## Declarations

### Author contribution statement

TBC.

### Funding statement

This work was supported by the Govan Mbeki Research Development Centre, University of Fort Hare, South Africa.

### Competing interest statement

The authors declare no conflict of interest.

### Additional information

No additional information is available for this paper.

## References

[bib1] Anderson J.A., Churchill G.A., Autrique J.E., Tanksley S.D., Sorrells M.E. (1993). Optimizing parental selection for genetic linkage maps. Genome.

[bib2] Animasaun D.A., Morakinyo J.A., Krishnamurthy R. (2015). Assessment of genetic diversity in accessions of pearl millet (*Pennisetum glaucum*) and napier grass ( *Pennisetum purpureum )*. Iran. J. Genet. Plant Breed..

[bib3] Bibi T., Talat M., Yasin M., Tariq M., Hasan E. (2013). Correlation studies of some yield related traits in linseed (*Linum usitatissimum* L.). J. Agric. Sci..

[bib4] Bosland P.W., Votava E.J. (2000). Peppers: Vegetable and Spice Capsicums.

[bib5] Chen S., Pang X., Song J., Shi L., Yao H., Han J., Leon C. (2014). A renaissance in herbal medicine identification : from morphology to DNA. Biotechnol. Adv..

[bib6] Dikshita N., Sivarajb N. (2015). Analysis of agromorphological diversity and oil content in Indian linseed germplasm. Fats and Oils.

[bib7] Garcia C.C., Barfuss M.H.J., Sehr E.M., Barboza G.E., Samuel R., Moscone E.A., Ehrendorfer F. (2016). Phylogenetic relationships , diversification and expansion of chili peppers. Ann. Bot..

[bib8] Ibarra-Torres P., Valadez-Moctezuma E., Pérez-Grajales M., Rodríguez-Campos R., Jaramillo-Flores M.E. (2014). Inter- and intraspecific differentiation of *Capsicum annuum* and *Capsicum pubescens* using ISSR and SSR markers. Sci. Hortic..

[bib9] Igwe D.O., Afiukwa C.A., Ubi B.E., Ogbu K.I., Ojuederie O.B., Ude G.E. (2017). Assessment of genetic diversity in *Vigna unguiculata* L . ( Walp ) accessions using inter-simple sequence repeat ( ISSR ) and start codon targeted ( SCoT ) polymorphic markers. BMC Genet..

[bib10] Ince A.G., Karaca M., Onus A.N. (2010). Genetic relationships within and between *Capsicum* species. Biochem. Genet..

[bib11] Jia X., Wang T., Zhai M., Li Y., Guo Z. (2011). Genetic diversity and identification of Chinese-grown pecan using ISSR and SSR Markers. Molecules.

[bib12] Karaca M., Onus A.N. (2010). Genetic relationships within and between *Capsicum*. Biochem. Genet..

[bib13] Maity S., Datta A.K., Chattopadhyay A. (2009). Seed protein polymorphism in nine species of Jute. Indian J. Sci. Technol..

[bib14] Mandal A., Datta A.K., Datta S., Gupta S. (2013). Genetic assessment of eight *Corchorus* spp . (Tiliaceae ) using RAPD and ISSR markers. Nucleus.

[bib15] Olatunji T.L., Afolayan A.J. (2019). Comparative quantitative study on phytochemical contents and antioxidant activities of *Capsicum annuum* L. and *Capsicum frutescens* L. Sci. World J..

[bib16] Olatunji T.L., Afolayan A.J. (2018). Contributions to the classification of *Capsicum annuum* L. and *Capsicum frutescens* L. in West Africa using morphological traits. Not. Bot. Horti Agrobot. Cluj-Napoca.

[bib17] Olatunji T.L., Morakinyo J.A. (2016). Pollen grain and hybridization studies in the genus *Capsicum*. Not. Sci. Biol..

[bib18] Olatunji T.L., Morakinyo J.A. (2015). Crude protein profiling of varieties of *Capsicum annuum* and *Capsicum frutescens* using SDS-PAGE. IOSR J. Pharm. Biol. Sci..

[bib19] Patel H.K., Fougat R.S., Kumar S., Mistry J.G., Kumar M. (2015). Detection of genetic variation in *Ocimum* species using RAPD and ISSR markers. 3 Biotech.

[bib20] Pfeiffer T., Roschanski A.M., PannellKorbecka G., Schnitter M. (2011). Characterization of microsatellite loci and reliable genotyping in a polyploidy plant, *Mercurialisperennis* (Euphorbiaceae). J. Hered..

[bib21] Prasad M.P. (2014). Molecular characterization and genetic diversity determination of *Hibiscus* species using RAPD molecular markers. Asian J. Plant Sci. Res..

[bib22] Rana M., Sharma R., Sharma P. (2014). Estimation of genetic diversity in *Capsicum annuum* L . germplasm using PCR-based molecular markers. Natl. Acad. Sci. Lett..

[bib23] Subramanyam K., Subramanyam K., Rajasekhar P., Reddy C.S. (2012). Assessment of genetic relationships among South Indian chilli ( *Capsicum annum* L .) cultivars using RAPD and ISSR Markers. Asian Australas. J. Plant Sci. Biotechnol..

[bib24] Sunar S., Yildirim N., Sengul M., Agar G. (2016). Genetic diversity and relationships detected by ISSR and RAPD analysis among *Aethionema* species growing in Eastern Anatolia ( Turkey ). Comptes Rendus Biol..

[bib25] Thul S.T., Darokar M.P., Shasany A.K., Khanuja S.P.S. (2012). Molecular profiling for genetic variability in *Capsicum* species based on ISSR and RAPD Markers. Mol. Biotechnol..

[bib26] Tong N., Bosland P.W. (2003). Observations on interspecific compatibility and meiotic chromosome behavior of *Capsicum buforum* and *C. lanceolatum*. Genet. Resour. Crop Evol..

[bib27] Tyagi A.K., Sharma M.K., Surya M.S.K., Kerkhi S.A., Chand P. (2014). Estimates of genetic variability, heritability and genetic advance in linseed (*Linum usitatissinum* L.) germplasm. Prog. Agric..

[bib28] Xiao-min Z., Zheng-hai Z., Xiao-zhen G., Sheng-li M., Xi-xiang L., Chadœuf J., Palloix A., Li-hao W., Bao-xi Z. (2016). Genetic diversity of pepper (*Capsicum* spp. ) germplasm resources in China reflects selection for cultivar types and spatial distribution. J. Integr. Agric..

